# The ER retention protein RER1 promotes alpha-synuclein degradation via the proteasome

**DOI:** 10.1371/journal.pone.0184262

**Published:** 2017-09-06

**Authors:** Hyo-Jin Park, Daniel Ryu, Mayur Parmar, Benoit I. Giasson, Nikolaus R. McFarland

**Affiliations:** 1 Center for Translational Research in Neurodegenerative Disease, Department of Neurology, College of Medicine, University of Florida, Gainesville, FL, United States of America; 2 Division of Biology of Aging, Department of Aging and Geriatric Research, College of Medicine, University of Florida, Gainesville, FL, United States of America; 3 Center for Translational Research in Neurodegenerative Disease, Department of Neuroscience, McKnight Brain Institute, College of Medicine, University of Florida, Gainesville, FL, United States of America; Louisiana State University Health Sciences Center, UNITED STATES

## Abstract

Abnormal accumulation of α-synuclein (αSyn) has been linked to endoplasmic-reticulum (ER) stress, defective intracellular protein/vesicle trafficking, and cytotoxicity. Targeting factors involved in ER-related protein processing and trafficking may, therefore, be a key to modulating αSyn levels and associated toxicity. Recently retention in endoplasmic reticulum 1 (RER1) has been identified as an important ER retrieval/retention factor for Alzheimer’s disease proteins and negatively regulates amyloid-β peptide levels. Here, we hypothesized that RER1 might also play an important role in retention/retrieval of αSyn and mediate levels. We expressed RER1 and a C-terminal mutant RER1Δ25, which lacks the ER retention/retrieval function, in HEK293 and H4 neuroglioma cells. RER1 overexpression significantly decreased levels of both wild type and A30P, A53T, and E46K disease causal mutants of αSyn, whereas the RER1Δ25 mutant had a significantly attenuated effect on αSyn. RER1 effects were specific to αSyn and had little to no effect on either βSyn or the Δ71–82 αSyn mutant, which both lack the NAC domain sequence critical for synuclein fibrillization. Tests with proteasomal and macroautophagy inhibitors further demonstrate that RER1 effects on αSyn are primarily mediated through the ubiquitin-proteasome system. RER1 also appears to interact with the ubiquitin ligase NEDD4. RER1 in human diseased brain tissues co-localizes with αSyn-positive Lewy bodies. Together, these findings provide evidence that RER1 is a novel and potential important mediator of elevated αSyn levels. Further investigation of the mechanism of RER1 and downstream effectors on αSyn may yield novel therapeutic targets for modulation in Parkinson disease and related synucleinopathies.

## Introduction

Parkinson disease (PD) is a neurodegenerative disorder that is associated with the progressive loss of dopamine cells in the substantia nigra that results in symptoms such as tremor, rigidity, and bradykinesia [1]. While the exact cause of this cell loss remains unknown, evidence suggests that both genetic and environmental factors contribute to a cascade of events that involve abnormal protein processing and accumulation, mitochondrial dysfunction, oxidative stress, excitotoxicity, inflammation, and other potential mechanisms [2–4]. In addition to dopamine loss, a major pathological hallmark of Parkinson’s disease and related disorders—including familial Parkinsonism, Lewy body dementia, and multiple system atrophy—is the presence of abnormal intracellular inclusion called Lewy bodies and neurites that are enriched with the protein, α-synuclein (αSyn) [5–8]. Targeting αSyn for therapeutics in PD is supported by multiple lines of evidence, including genetic mutations and multiplications in the gene, *SNCA*, that are associated with rare forms of familial parkinsonism [9,10]. In animal studies, overexpression of human αSyn results in Lewy body-like accumulations, degeneration of nerve terminals and locomotor defects [11–13] and such changes can be reversed when the transgene is switched off [14]. Conversely, knockout of αSyn in mice confers protection against certain toxins [15]. Therefore, one strategy for PD therapeutics has focused on promoting the degradation of αSyn and reducing its basal level in neurons.

Cells have an extensive cellular protein quality control network, which ensure that proteins fold correctly and, if misfolded or damaged that they are cleared from the cell [16]. Cellular protein degradation machinery is highly complex and modulated by many different factors, including proteins involved in intracellular trafficking. Members of the Rab GTPase protein family, as well as Arf and RalB GTPases, are well-known regulators of membrane trafficking and fusion events, and play key roles in the regulation of the autophagic process [17]. Ubiquitination not only targets proteins for degradation, but also regulates protein trafficking, involving endosomes, and other functions [18].

Recently, we and other groups have identified human Retention in endoplasmic reticulum 1 (RER1) as an important protein that mediates ER-Golgi trafficking of Alzheimer’s disease (AD)-related proteins and significantly decreases amyloid-β (Aβ) production [19–21]. Mammalian Rer1 has also been reported to regulate other proteins such as muscular acetylcholine receptors [22] and Foxj1a [23], suggesting important roles in neuromuscular synapses and ciliogenesis.

RER1 was first identified in yeast as the ER retention factor of Sec12p, Sed4p, Mns1p, Sec71p, and Sec63p [24–27]. Yeast Rer1 binds to transmembrane domains (TMDs) of these proteins and carries them back to the ER via COPI vesicles [26,28]. In addition to Rer1, yeast Bsd2 appears to have a similar role as a TMD-dependent ER retention factor and indeed, they share substrates [29]. Interestingly, Bsd2 is known to act as a specific adaptor linking the ubiquitin ligase NEDD4 ortholog, Rsp5, to its substrates and contributes to the ubiquitination and sorting of membrane proteins into the multivesicular body (MVB) pathway, suggesting a possible role for Rer1 in protein ubiquitination, trafficking, and degradation. It has been also reported that RER1 degradation is mediated by synoviolin, a member of the E3 ubiquitin ligase family, and synoviolin affects RER1-mediated transport and quality control of RER1 substrates by regulating RER1 levels [30,31].

Although αSyn was initially identified as a synaptic vesicle-associated protein, recent data indicate that αSyn accumulates within the ER [32,33] and causes toxicity in a mouse model of synucleinopathy [34,35]. In addition, both the ER-Golgi transition and integrity of the Golgi apparatus are known to be sensitive to the overexpression of αSyn [36,37]. αSyn-related ER- Golgi vesicle trafficking defects have been rescued by overexpression of the Rab GTPase, Rab1, that regulates ER to Golgi protein trafficking [37], indicating that modulation of protein transport and homeostasis can abrogate αSyn toxicity. As RER1 appears also to play an important role in ER-Golgi trafficking and modulation of proteins, we hypothesized that RER1 might also play an important role in regulation of trafficking and accumulation of toxic forms of αSyn. In this study we examine the effect of RER1 expression on αSyn in human and neuronal cell lines, and its expression pattern in Lewy body disease tissue. Here we report the first evidence that RER1 expression significantly reduces αSyn and may regulate levels through induction of the UPS pathway.

## Materials and methods

### Constructs

Construction of pcDNA3.1 vector used to express human wild-type, A30P, A53T, E46K, or Δ71–82 αSyn (GenBank L08850) was as previously described [38–40]. The αSyn K80R mutant was generated by site-directed mutagenesis using wild-type αSyn. Human wild-type RER1 construct with or without myc tag was previously described [19]. RER1Δ25 construct was generated by PCR using pAG3zeo wild-type RER1. The constructs of HA-NEDD4 (Plasmid #27002) and EGFP-LC3 (Plasmid #11546) were purchased from Addgene.

### Cell cultures and transfections

Human embryonic kidney 293 (HEK293) cells were cultured at 37°C in a humidified 5% CO_2_ incubator in Dulbecco’s modified Eagle’s medium (DMEM) containing 10% fetal bovine serum (FBS). Human neuroglioma H4 cells were cultured in OptiMEM containing 5% FBS. Cells were grown to 60–80% confluency and transfected with Lipofectamine 2000 (Life Technologies) or Superfect (Qiagen) in accordance with the manufacturer’s instructions. Cells were harvested 36 h after transfection for analysis.

To generate αSyn stable lines, H4 cells were transfected with human αSyn cDNA and pBLAST (Invitrogen) using Superfect. Cells were selected with blasticidin at 3 μg/ml (Invivogen) and screened by western blot with αSyn antibody.

### Inhibition of protein degradation pathways

24 h after transfection, cells were treated with 10 μM MG132 (Sigma), 100 μM Chloroquine (Sigma), 100 nM Bafilomycin (Millipore), or 10 μM Eeyarestatin1 (Sigma). 10–12 h after treatment, cells were harvested for analysis. To test the activity of Chloroquine, 10, 50, or 100 μM Chloroquine was treated into HEK293 cells for 12 h. To visualize LC3-II accumulation, HEK293 cells were transfected with EGFP-LC3 and 24 h after transfection cells were treated with 100 μM Chloroquine for 12 h.

### Western blot

Cell lysis and Western blotting were performed as described previously [19,41]. Detergent lysates of cells were prepared using RIPA buffer (50 mM Tris (pH 7.4), 150 mM NaCl, 5 mM EDTA, 0.5% Nonidet P-40, and 0.5% sodium deoxycholate) supplemented with protease inhibitors (a mixture of 4-(2-aminoethyle) benzenesulfonyl fluoride, pepstatin A, E-64, bestain, leupeptin and aprotinin; Sigma). For sequential extractions, cells were lysed with 1% Triton X-100 in PBS and then the pellets were resuspended and lysed in 2% SDS. The bicinchoninic acid (BCA) assay was performed to quantitate total protein in accordance with the manufacturer’s instructions (Millipore). Lysates were subjected to 4–2% Bis-Tris SDS-PAGE and transferred to a nitrocellulose membrane prior to incubation with selected antibodies. Immunoreactive bands were visualized using an Odyssey infrared scanner (LiCor Biosciences).

The antibodies used in this study were αSyn (610786, BD); αSyn (AB5038; Millipore); pSer129-αSyn (81A)[42]; PAN-Syn (AB6176, Abcam); RER1 (AP-R76) [19] and (R4407 for endogenous RER1, Sigma); β-actin (AC-15, Sigma); GAPDH (14C10, Cell Signaling); Flag (M2, Sigma); HA (H3663, Sigma); GM130 (610823, BD); KDEL (10C3, Stressgen); APP (A8717, Sigma); EGFP (A11122, Life Technologies).

### Co-immunoprecipitation

Subconfluent dishes of stable cell lines were washed twice with ice-cold PBS and solubilized in CHAPS Co-IP buffer (1% CHAPS (Calbiochem), 50 mM Tris, pH 7.4, 150 mM NaCl, 5 mM EDTA) supplemented with protease inhibitors. Lysates were subject to centrifugation at 13,200 rpm for 10 min at 4°C, and the resulting supernatant was used for co-immmunoprecipitation with respective antibodies at 4°C overnight. The immune complexes were collected with Protein A-conjugated agarose beads (Pierce) and eluted by incubation at 50°C for 15 min or 100°C for 5 min in the SDS sample buffer. The resulting immunoprecipitates as well as detergent lysates corresponding to 2% of the volume used for immunoprecipitation were resolved by 4–2% Bis-Tris SDS-PAGE and analyzed by western blot with the indicated antibodies.

### Immunohistochemistry and immunofluorescence analysis

Paraffin-embedded human brain tissue sections (2 men and 1 woman for both LB positive and negative cases) were deparaffinized and hydrated through a series of graded ethanol solutions followed by PBS. The sections were incubated in Citrate buffer (10 mM Trisodium citrate, 0.05% Tween-20, pH6.0) at 90°C for 10 min for antigen retrieval. After washing in PBS, tissues were incubated in PBS-T (0.3% Triton X-100 in PBS) for 10 min. Tissues were blocked in 5% normal goat serum for 30 min and incubated in primary antibody at 4°C overnight. Immunostaining was visualized with biotinylated secondary, followed by avidin-biotin (Vectastain Elite Kit, Vector Labs), and 3,3’-diaminobenzidine reaction as previously described [43]. For immunofluorescence analysis, immunostaining was visualized with either fluorescent secondary (1:250 dilution; Alexa Fluor 488 or 594, Molecular Probes). For immunofluorescence staining of cultured cells, cells were grown and transfected on chamber slides. After antigen retrieval, cells were stained using same methods as tissue staining. Slides were scanned using an AperioScan Scope CS (40×magnification; AperioTechnologies Inc.) and fluorescence images of representative areas were acquired on EVOS FL Digital Microscope (EMS), Olympus IX81-DSU Confocal Microscope (Olympus), or TCS SP2 AOBS Spectral Confocal Microscope (Leica).

### Statistical analyses

All data were expressed as group mean ± SEM. Data were graphed as means with standard error using GraphPad Prism 5.0 software (GraphPad, La Jolla). Data from optical density measurements from Western blots were analyzed using one-way ANOVA with Tukey’s multiple comparisons test where applicable.

## Results

### RER1 overexpression decreases αSyn levels

**Fig 1:**
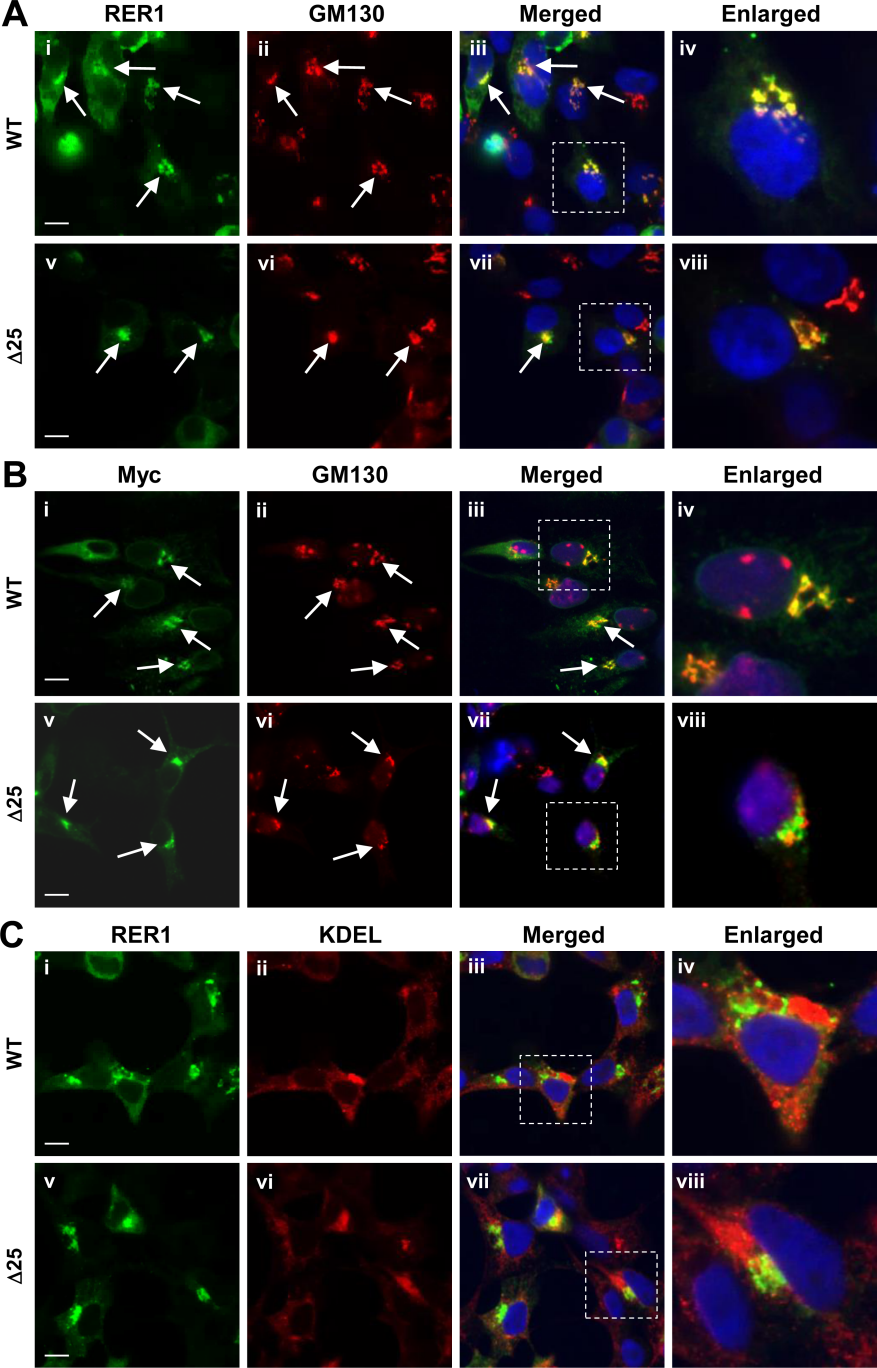
RER1 localizes in cis-Golgi network. HEK293 cells were transfected with RER1 or the mutant RER1Δ25 (untagged vs myc-tagged, panels A vs B, respectively) and stained with RER1 and GM130 antibodies (A), myc and GM130 antibodies (B), or RER1 and KDEL antibodies (C). All photos were acquired with an Olympus IX81-DSU Confocal Microscope. The dotted box indicates the area enlarged (in panels iv and viii). (A, B) Perinuclear wild type RER1 immunoreactivity (green) are colocalized with GM130 (red), a cis-Golgi matrix protein. In contrast, perinuclear RER1Δ25 immunoreactivity only partially merges with GM130. Arrows indicate cells with colocalization. (C) Vesicular patterns of both wild type RER1 and RER1Δ25 immunoreactivities (green) do not merge KDEL proteins (red), an ER marker. Scale bar = 10 μm.

To determine the effects of RER1 on αSyn levels, we utilized pAG3zeo construct to overexpress RER1 in HEK293 and H4 cells. We tested also RER1Δ25, a C-terminal deletion mutant that has previously been shown in yeast to affect its localization, binding with the COPI coatomer, and function [20,44,45]. Immunofluorescence assay (IFA) with wild type RER1 (RER1 wt) and RER1Δ25 in HEK293 cells demonstrated that RER1 wt colocalized with the cis-Golgi marker GM130 (Fig 1A and 1B, panel iv), whereas the untagged and myc-tagged RER1Δ25 only partially colocalized with GM130 (Fig 1A and 1B, panel viii). Neither RER1 nor RER1Δ25 colocalized with the ER marker, KDEL (Fig 1C). These findings indicate that human RER1 requires the C-terminal 25 amino acids for correct localization in early secretory compartments and that RER1Δ25 may have altered function.

**Fig 2:**
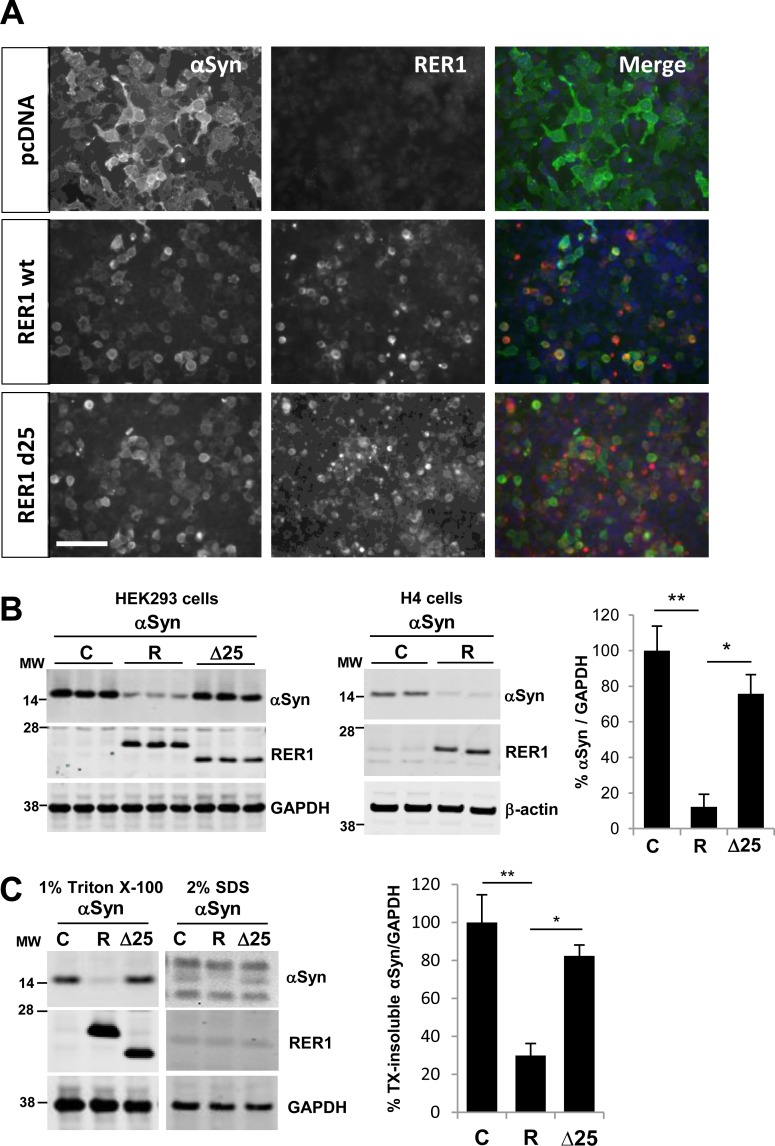
RER1 expression significantly reduces αSyn levels. (A) αSyn and RER1 were transiently overexpressed in HEK293. Cells were co-stained with αSyn (green) and RER1 (red) specific antibodies. The photos show that αSyn-immunoreactivity was reduced by wild type RER1 and less so by mutant RER1Δ25 expression. (B) Wild type RER1 expression significantly decreased αSyn levels (mean, 87.8 ±7.1%), but expression of RER1Δ25 resulted in significantly smaller effects on levels of αSyn (mean, 24.3 ±10.6%) (*p<0.05, **p<0.01; F_2,6_ = 17.31, p = 0.0032) (n = 5/control, n = 7/RER1, n = 3/ RER1Δ25) in HEK293. RER1 also decreased αSyn levels in human neuroglioma H4 cells (middle panel). (C) αSyn levels in both 1% Triton X-100 and 2% SDS fractions were decreased by RER1 overexpression (*p<0.05, **p<0.01; F_2,9_ = 14.02, p = 0.0017) (n = 4/group). C = control; R = RER1 transfected; Δ25 = RER1Δ25. Scale bar is 40 μm.

Next we sought to determine the effects of RER1 and RER1Δ25 expression on αSyn levels. IFA using an αSyn specific antibody showed a marked decrease in αSyn-positive cells when co-expressed with RER1 (Fig 2A). Western blot of whole cell lysates confirmed that overexpression of RER1 caused a significant reduction (87.8 ±7.1%, p<0.005) in the level of αSyn at 24–36 h post-transfection relative to control (Fig 2B). By contrast, RER1Δ25 expression showed only partial reduction (24.3 ±10.6%, p<0.01) of αSyn levels, consistent with an altered function and potential disruption of COP1 coatomer binding that may be necessary for its effects on αSyn (Fig 2B). Separation of cell lysates into Triton X-100 (1% TX) soluble and insoluble (solubilized in 2% SDS) fractions further demonstrated that RER1 expression significantly reduced TX-soluble αSyn compared to control and RER1Δ25 expressing groups (Fig 2C). RER1 is not detected in insoluble fractions by western blot and only small fractions of αSyn is detected in the fractions. However, insoluble αSyn is also reduced by RER1 co-expression (Fig 2C, right panel), suggesting an RER1 effect on insoluble αSyn aggregates.

### Effects of RER1 are specific to αSyn

**Fig 3:**
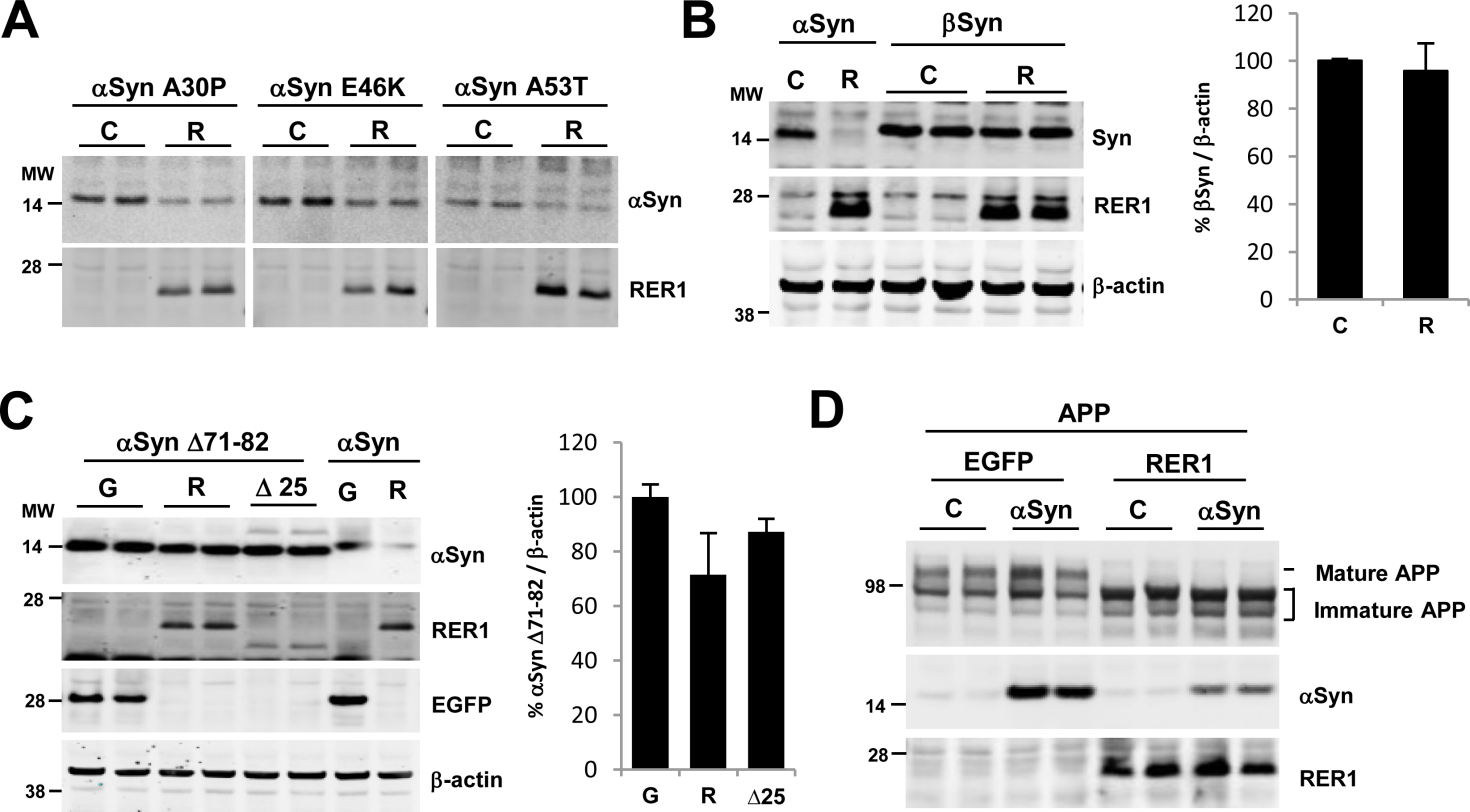
RER1 effects are specific to αSyn. (A) RER1 overexpression decreased the levels of αSyn mutants (A30P, E46K, and A53T). (B) The levels of βSyn did not change with RER1 overexpression (p = 0.725) (n = 4/group). (C) Expression of αSyn Δ71–82 mutant which is unable to aggregate due to the lack of a corresponding middle hydrophobic region, is not significantly decreased by RER1 overexpression (F_2,15_ = 2.214, p = 0.1438) (n = 6/group). (D) Overexpression of αSyn does not affect the maturation of APP or RER1 retrieval/retention function. C = control; R = RER1 transfected; G = EGFP

In addition to wild type (WT) αSyn, we tested whether RER1 could decrease the levels of mutant forms αSyn causal of human disease. Expression of RER1 in HEK293 cells decreased the levels of A30P, E46K, and A53T αSyn mutants (Fig 3A). We next tested whether the effects of RER1 expression were specific to αSyn. βSyn exhibits high sequence homology and structural similarity with αSyn; however, in contrast to αSyn, RER1 overexpression had no effect on βSyn levels (Fig 3B). Since a major difference between αSyn and βSyn is the hydrophobic non-amyloid beta (Aβ)-component (NAC) region responsible for the ability of the protein aggregation and fibrillization, we hypothesized that RER1 effects on αSyn might require the intact NAC sequence. Therefore, we tested the effects RER1 expression against the αSyn mutant, delta 71–82 (αSyn Δ71–82), which lacks intact NAC domain sequence critical for αSyn fibrillization. Similar to βSyn, RER1 expression had no effect on αSyn Δ71–82 levels, suggesting that RER1 effects on αSyn are dependent on the hydrophobic core of the NAC region (Fig 3C). However, how RER1 interacts with αSyn (and its NAC domain) remains unclear. In co-IP assays we were unable to demonstrate association of RER1 with αSyn, indicating that αSyn is unlikely to be direct substrate of RER1 (see **S1** Fig).

As shown in Figs 2B and 3C, the expression levels of RER1Δ25 tended to be lower than RER1 wt. This was not unexpected because it has been shown that RER1Δ25 is targeted to the vacuole via the multivesicular body (MVB) sorting pathway, suggesting a rapid processing of the mutant Rer1 [44]. To rule out the possibility that αSyn expression itself is affected by the saturated protein synthesis machinery due to co-expression, we also co-expressed enhanced green fluorescence protein (EGFP) with αSyn and compared the protein levels with RER1 expressing samples. In Fig 3C, high levels of EGFP expression did not decrease the levels of αSyn and αSyn Δ71–82.

### α-Synuclein expression does not influence RER1 or APP processing

We have previously shown that RER1 attenuates the trafficking of APP and γ-secretase With co-expression of RER1 and APP, RER1 significantly reduced mature APP and increased immature APP. Live staining of APP also showed that the levels of APP on plasma membrane was significantly reduced although total levels of APP was not changed [19]. To determine whether accumulated αSyn has an effect on RER1’s trafficking function, we co-expressed RER1 and APP with or without αSyn in HEK293 cells and analyzed the levels of mature and immature APP. As expected RER1 overexpression significantly decreased mature APP and increased immature APP (Fig 3D), indicating that APP trafficking from early secretory compartment to late secretory compartment is delayed by RER1. αSyn overexpression did not affect APP maturation in the presence or absence of RER1 (Fig 3D, top panel). Likewise, APP overexpression did not alter RER1 effects on αSyn levels (Fig 3D, middle panel). These findings argue that αSyn does not influence APP trafficking or RER1 function, though do not exclude the possibility that RER1 effects on αSyn and APP are independent of each other.

### RER1-mediated αSyn degradation is dependent on ubiquitin proteasome system

**Fig 4:**
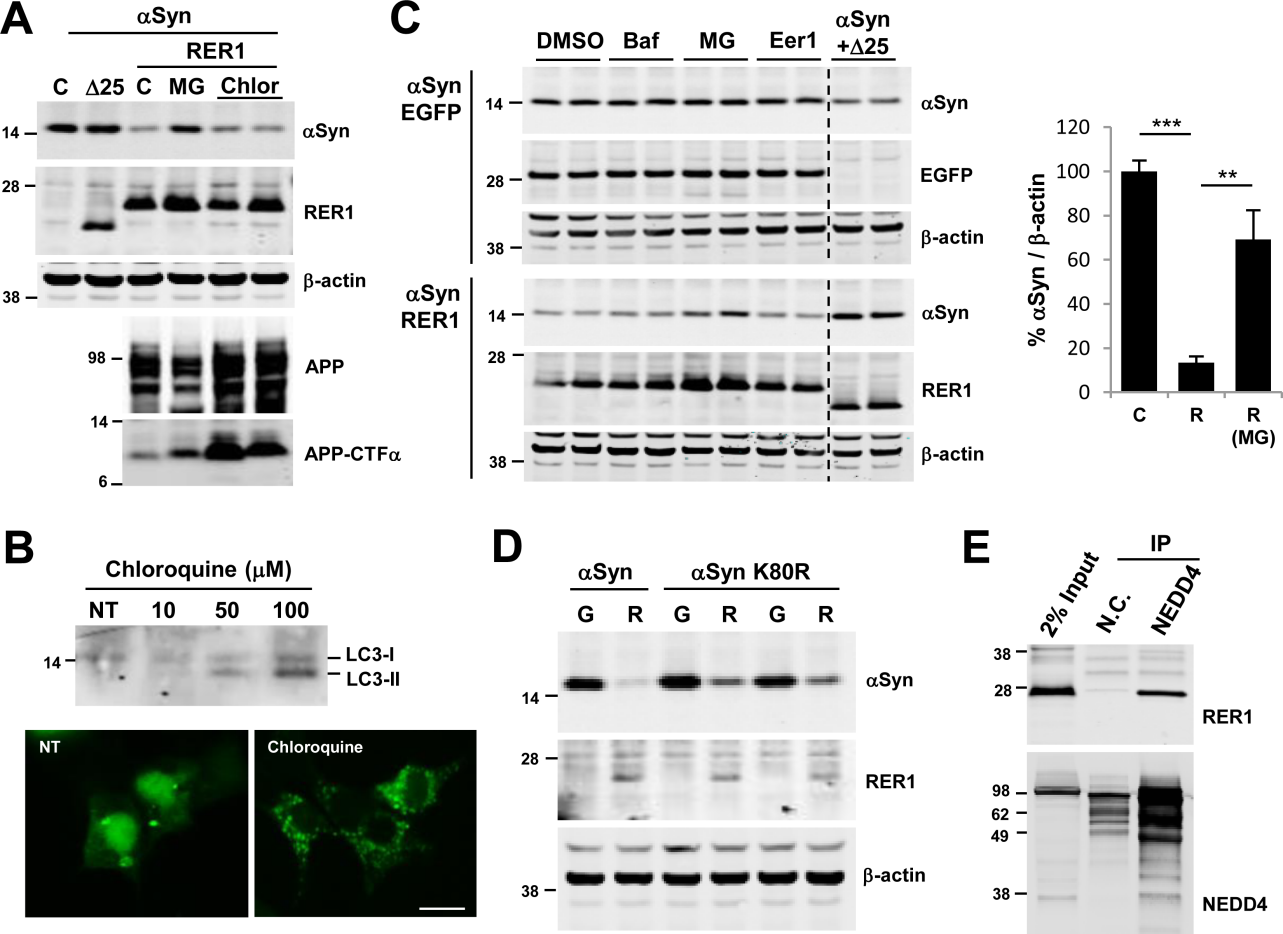
Proteasome inhibition rescues αSyn levels. (A) 24h post transfection, cells were treated with 10 μM MG132 (MG) or 100 μM chloroquine (Chlor). MG132 treatment partially recovered RER1-mediated reduction of αSyn. In contrast, chloroquine did not rescue αSyn, but increased APP levels consistent with its effects on macroautophagy and the lysosome. (B) Chloroquine treatment blocks autophagy activity. Cells were treated with 10–100 μM Chlorquine for 24 h. Lipidated and sequesterted LC3-II increased by chloroquine treatment (top panel). Cells were transfected with GFP-LC3 and 24 h post transfection, cells were treated with 100 μM Chloroquine for 24 h. Diffused pattern of LC3-I decreased and punctated pattern of LC3-II increased by Chloroquine treatment (bottom panel; scale bar = 10 μm). (C) Cells were co-transfected with αSyn and either EGFP or RER1, and then 24h post transfection treated with DMSO, 100 nM Bafilomycin (Baf), 10 μM MG132 (MG), or 10 μM Eeyarestatin1 (Eer1). In cells co-transfected with EGFP control, MG132 did not increase αSyn levels compared to cells exposed to DMSO. In cells co-transfected with RER1, MG132 showed a similar partial recovery of RER1-mediated αSyn reduction (mean, 69.2%), whereas the macroautophagy and ERAD inhibitors, Bafilomycin and Eeyarestatin1, had no apparent effect (**p<0.01, ***p<0.001; F_2,6_ = 28, p = 0.0009; n = 3/group). The right 2 lanes (separated by dotted line) show lysates from cells co-transfection of αSyn with RER1Δ25 for comparison. (D) RER1 decreases the levels of αSyn K80R mutant significantly. αSyn wild type or K80R mutant was co-transfected with RER1 or EGFP into HEK293 cells. R = RER1 transfected; G = EGFP. (E) RER1 interacts with NEDD4. RER1 is co-immunoprecipitated with NEDD4 in HEK293 cells co-expressing RER1 and NEDD4. N.C. = negative control using nonreactive serum.

To determine the mechanism by which RER1 reduces αSyn levels, we used selective inhibitors of the proteasome, macroautophagy, and ER associated degradation (ERAD). MG132 (N-(benzyloxycarbonyl)-leucinyl-leucinyl-leucinal) is a potent, reversible inhibitor of the 20S proteasome and effectively blocks the proteolytic activity of the 26S proteasome complex. In the presence of MG132, RER1-mediated reduction in αSyn levels was partially recovered (Fig 4A). However, using the macroautophagy inhibitor, chloroquine, we did not observe any rescue of αSyn levels due to RER1-coexpression. Block of macroautophagy by chloroquine was confirmed by accumulation of the marker LC3-II (Fig 4B). Full length-amyloid precursor protein (APP) as well as a C-terminal fragment of APP were accumulated by chloroquine treatment, suggesting it blocks fusion of autophagosomes as well as late endosomes (Fig 4A) [46,47]

To test whether the proteasome inhibitor blocked general degradation of αSyn or RER1-specific degradation of αSyn, we also treated cells with EGFP and no RER1 overexpression. MG132 did not increase αSyn levels in cells without RER1 overexpression, compared to DMSO treated controls (Fig 4C, left upper panel). However, MG132 treatment increased αSyn levels in RER1 overexpressing cells (69.2 ±13.1%, p<0.01) (Fig 4C, left bottom and right panel), suggesting that RER1-mediated αSyn degradation may occur in part through the ubiquitin-proteasome system (UPS). We also tested if another macroautophagy inhibitor, bafilomycin (Baf), or ER-associated degradation (ERAD) inhibitor, eeyarestatin1 (Eer1), could block the RER1 effects on αSyn, but none of these significantly changed the protein levels (Fig 4C, left panel). Since αSyn has only one lysine in the NAC region at amino acid residue 80, we tested if this lysine (as a potential ubiquitination site) is important for RER1 to recognize αSyn for regulating its levels. However, RER1 also significantly decreased the αSyn K80R mutant (Fig 4D).

### RER1 interacts with NEDD4

In human cells, the HECT (homologous to the E6-AP carboxyl terminus) domain NEDD4 (neuronal precursor cell-expressed, developmentally down-regulated gene 4) E3 ligases have been shown to enhance ubiquitination and clearance of αSyn [48]. In yeast, the Nedd4 ortholog Rsp5 also regulates αSyn degradation, inclusion formation, and toxicity [48,49]. To determine whether RER1-dependent αSyn degradation is relevant to NEDD4, we performed co-immunoprecipitation (IP) studies of RER1 and NEDD4 in HEK293 cells. RER1 co-immunoprecipitated with NEDD4, whereas an unrelated antibody did not co-immunoprecipitate RER1 (Fig 4E). These findings suggest that RER1 interacts with NEDD4 and may regulate αSyn levels through a NEDD4-dependent degradation pathway.

### RER1 colocalizes with αSyn in Lewy bodies

**Fig 5:**
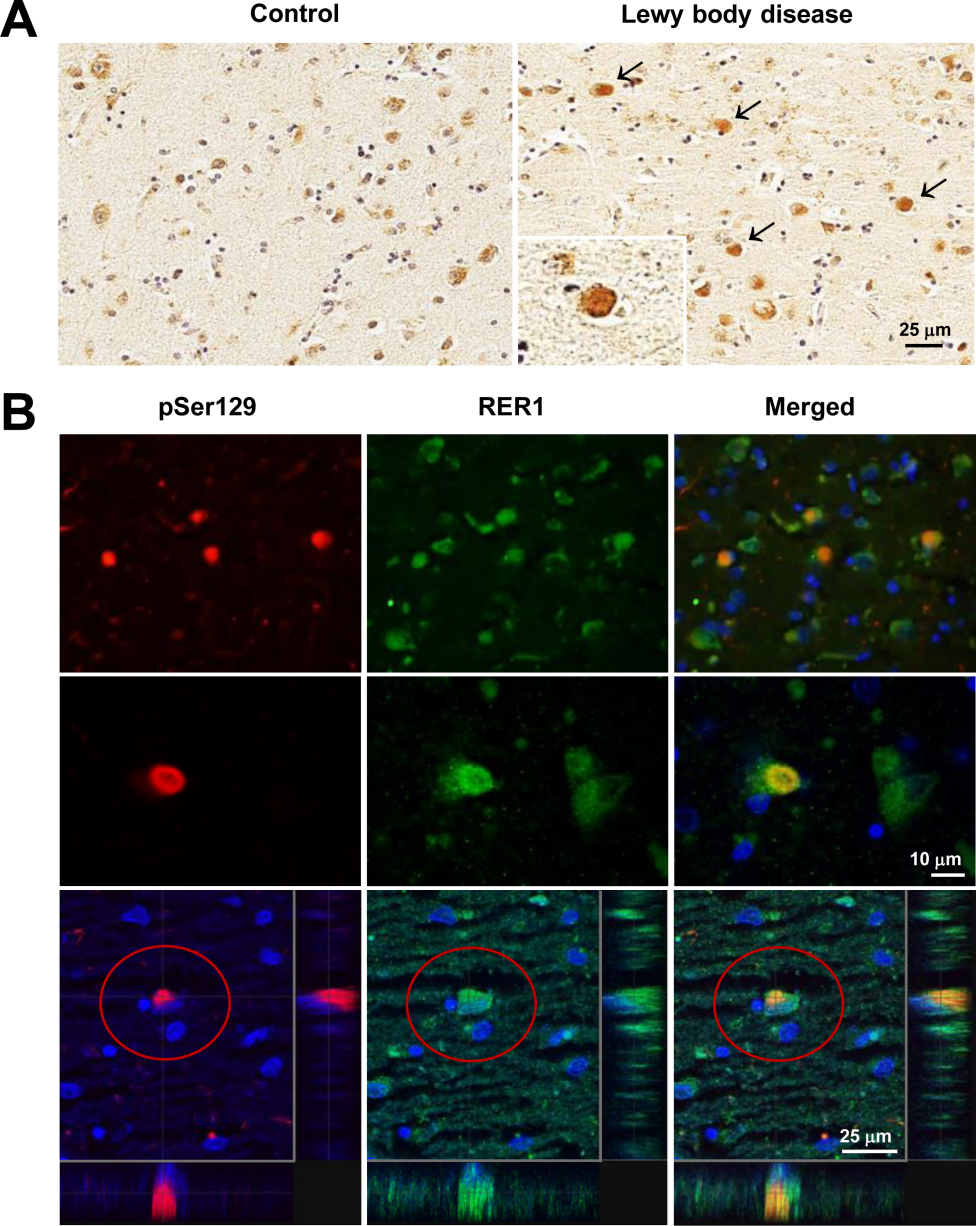
RER1 colocalizes with αSyn in Lewy bodies. (A) Photomicrogaphs of tissue from control (left), and LB-positive tissues (right) show that RER1 colocalizes with αSyn in Lewy bodies. RER1 is detected in cell bodies, but appears enriched in round LB-like inclusions (arrows; see also enlargement in inset). (B) Photos show colocalization of both RER1 (green) and phosphorylated αSyn (pSer129: red) immunofluorescence in round, LB-like structures. Below are higher power images and confocal mapping of an inclusion positive for both RER1 and pSer129 immunoreactivity. Images were acquired on TCS SP2 AOBS Spectral Confocal Microscope (Leica) (B).

Several proteins related to αSyn degradation or refolding have been found to colocalize with αSyn in LBs and Lewy neurites (LNs) [50–52]. In this study, we examined the distribution of RER1 in healthy human control and LB-positive disease brains (from Dementia with Lewy body cases) using an affinity purified RER1 antibody (AP-R76) [19]. In the healthy cortex, RER1 staining was seen primarily in cell bodies (Fig 5A, left). In contrast, RER1 staining was altered in the brain of LB positive patients. In addition to localization in the cell body, LB-like round structures were observed (Fig 5A, right, arrows). Double immunofluorescence for both RER1 and pSer129 αSyn and high-resolution confocal microscopy demonstrated co-localization of RER1 with LBs (Fig 5B).

## Discussion

Multiple lines of evidence indicate that abnormal accumulation and deposition of αSyn contributes to the development of LB disorders such as PD. Elucidating the mechanisms that regulate αSyn levels may be key to understanding the pathophysiology of the neurodegenerative disease and development of novel therapeutics. In this study, we present novel data that suggest RER1 is a mediator of UPS-dependent αSyn degradation. In human embryonic kidney and neuroglioma cell lines, RER1 overexpression significantly decreased levels of both wild type and PD-linked mutant forms of αSyn. These effects were specific to αSyn and not βSyn, or mutant Δ71–82 αSyn, suggesting that this stretch of hydrophobic residues is required for RER1-mediated decrease in αSyn. Furthermore, the effect of RER1 was blocked by inhibition of UPS, but not macroautophagy. We demonstrate also that RER1 accumulates and colocalizes with LBs in human DLB (dementia with Lewy body) brain tissue.

RER1 is an integral membrane protein that has four putative transmembrane domains predicted to form an M-shaped topology with both N- and C-termini facing the cytosol. RER1 localizes to early secretory compartments and actively recycles between the early Golgi and the ER (Fig 1) [20,53]. Considering its topology and localization, it is likely that RER1 interacts with both cytosolic and ER/Golgi transmembrane proteins. Previous reports have established RER1 as a limiting factor in trafficking, quality control, and multiprotein complex assembly of RER1 substrates in the early secretory pathway [19–22,54]. We have previously shown that RER1 modulates amyloid-β (Aβ) production by altering trafficking of γ-secretase and amyloid precursor protein (APP) in early secretory compartments [19]. Manipulation of RER1 levels showed effects on the maturation and trafficking of γ-secretase, but not total levels of the protease complex [19]. RER1 has been shown also to control total and surface levels of muscle acetylcholine receptor (AChRα) and rhodopsin by localizing immature substrates to the early secretory pathway [22,54]. Interestingly, our data suggest that correct subcellular localization of RER1 to the early secretory compartment including cis-Golgi is required for the regulation of αSyn levels (Figs 1 and 2). This finding implies that the RER1-dependent Golgi-ER-retrieval system is important not only for protein quality control and trafficking, but may also regulate degradation of certain proteins, such as αSyn.

**Fig 6:**
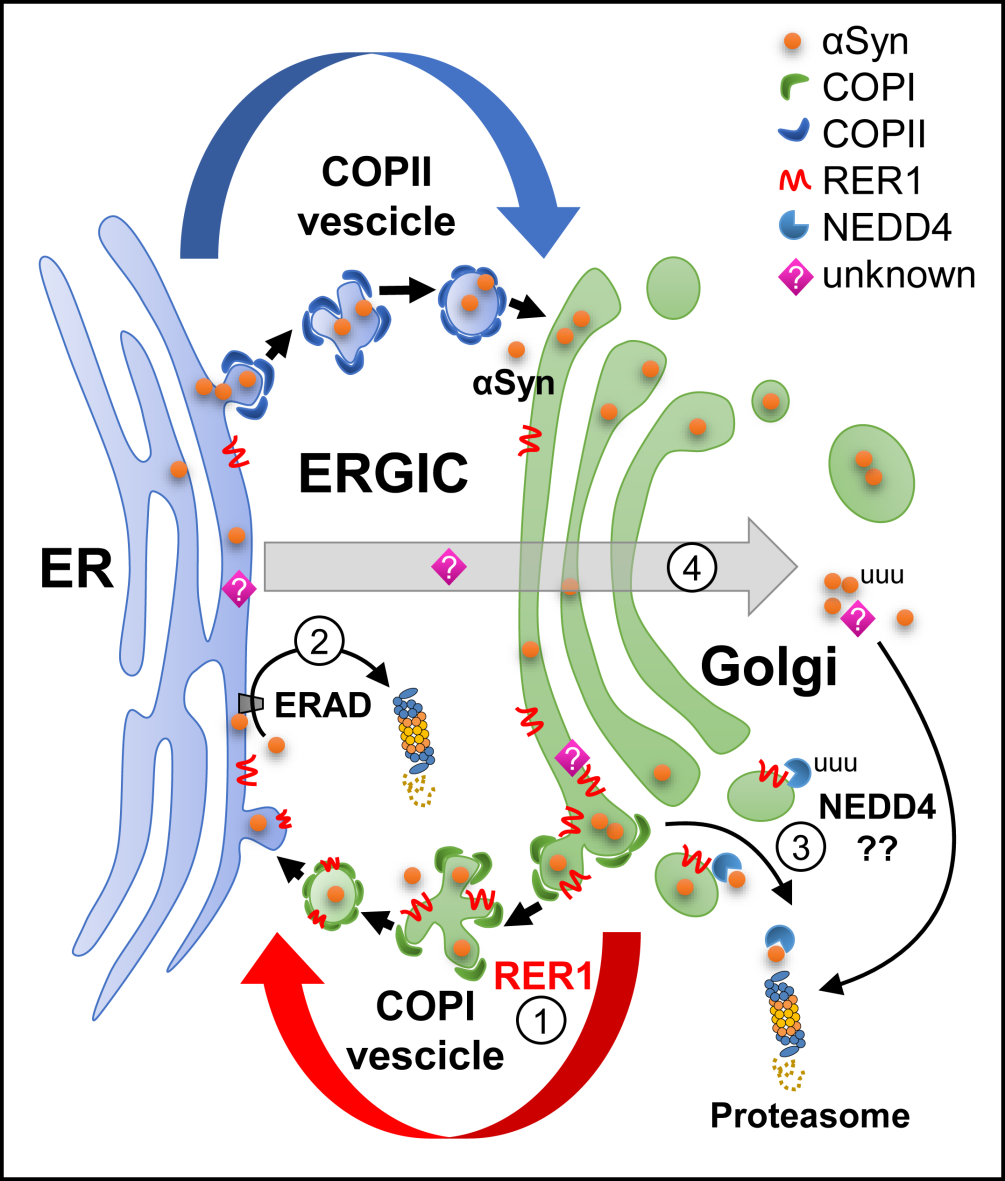
Summary and putative model of RER1 effects on αSyn. 1) RER1 expression increases ER retrieval/retention of “immature” proteins in the cis-Golgi compartment which may contribute to ER retention of αSyn. 2) RER1 may indirectly retrieve αSyn back to the ER for degradation via the ERAD and proteasome (unfolded response system). 3) NEDD4 was found to interact with RER1. Although an E3 ligase, NEDD4 has been shown to reduce αSyn though the endosomal-lysosomal pathway [48]. RER1-mediated degradation of αSyn may also occur independent of ubiquitin via the 20S proteasome. 4) RER1 expression may act though maturation of an unknown protein (?) that mediates targeting and disposal of excess cytosolic αSyn via the proteasome (through an ubiquitin independent mechanism). ER, endoplasmic reticulum; ERAD, ER-associated degradation; ERGIC, ER-Golgi intermediate compartment.

Accumulation and aggregation of αSyn is correlated with PD pathogenesis. Although αSyn localizes to the cytosol as a freely diffusing, unstructured protein, it possesses membrane-binding characteristics that allow interactions with presynaptic vesicles, implicating it in the regulation of the storage, exocytosis, and release of neurotransmitters [55–58]. Structural studies of different αSyn states, in the absence or presence of membranes, reveal that the interaction of αSyn with membranes may also contribute to abnormal aggregation and toxicity [57]. Accumulation of αSyn affects the ER to Golgi trafficking in a dose-dependent manner through the inhibition of vesicle docking or fusion to Golgi membranes [59]. In both human and mouse α-synucleinopathies, there is preferential accumulation of toxic αSyn oligomers within the ER/microsomes [34,35], suggesting that ER accumulation of αSyn may contribute directly to chronic ER stress. Considering the nature of RER1 and localization of toxic αSyn species in the ER [34,35] and intracellular vesicles [60], we posit that RER1 may retain or retrieve αSyn in/on early secretory compartments and facilitate transfer to protein degradation systems including the UPS and possibly also ERAD (Fig 6). Alternatively, it is also possible that RER1 decreases cytosolic αSyn which comprises the majority of αSyn intracellular levels. In an overexpression system, the immunostaining pattern of αSyn is diffuse and cytosolic (Fig 2A, left panels), and RER1 overexpression significantly decreased the diffused αSyn (Fig 2A and 2B). RER1 may regulate a downstream molecule that mediates the degradation of excess cytosolic αSyn (Fig 6).

Several reports indicate that the NAC region (non-amyloid-β component; amino acids 61–95) of αSyn is a critical determinant of the fibrillation process [61–63] and penetration of αSyn into micelles [64]. In our study, the effects of RER1 were significantly attenuated when RER1 was co-expressed with αSyn Δ71–82, which lacks 11 amino acids of the hydrophobic NAC region (Fig 3C). Similarly levels of βSyn, which also lacks this critical NAC domain, were unaffected by RER1 overexpression, whereas PD-linked mutants with N-terminal domain missense mutations (A30P, A53T, E46K) all showed comparable reductions (Fig 3A and 3B). These findings suggest that RER1 effects on synuclein are specific to αSyn and require an intact NAC region, important for fibrillization. Further studies are needed to determine whether insoluble, fibrillar forms of αSyn are preferentially targeted for RER1-mediated degradation.

Nearly all major degradation pathways have been implicated in αSyn degradation resulting in some controversy [65–72]. The UPS degrades αSyn independent of the pre-existing αSyn burden, whereas degradation of αSyn via macroautophagy appears to occur primarily when αSyn levels are abnormally increased such as that in transgenic αSyn mice [73]. Our inhibitor studies demonstrate that RER1-dependent αSyn degradation is mediated by the proteasome. Among several inhibitors, only the proteasome inhibitor, MG132, rescued αSyn levels, while treatment with autophagy/lysosomal inhibitors had no effect on RER1-dependent decrease of αSyn in the cell lines tested (Fig 4). We also examined whether RER1-dependent αSyn degradation may involve the ERAD by treatment with the ERAD inhibitor, Eer1, which directly binds p97/VCP ATPase that is critical for ERAD function [74]. Eer1 treatment had no effect on αSyn levels (Fig 4C), suggesting that RER1 effect on αSyn levels may be independent of ERAD. These findings, however, are preliminary and likely limited by dose and potential non-specific effects of Eer1. Moreover, inhibition of the ERAD, like macroautophagy, may result in compensatory increase in UPS function further mitigating change in αSyn levels [75].

In addition to αSyn, LBs commonly co-contain several other proteins associated with protein processing, including chaperone-related proteins [76,77], heat shock proteins [50,51], and ubiquitin degradation pathway-related proteins [69,78–82] among others. In this study we demonstrate that RER1 co-localizes with phosphorylated αSyn in LBs in brain tissue from DLB patients (Fig 5). These findings are the first to show that RER1 accumulates in LBs and support the notion that RER1 may play a role in processing abnormal αSyn aggregates/deposits in synucleinopathies such as LBD and PD. These preliminary findings, though intriguing, require further confirmation. It remains unclear how RER1 facilitates the degradation of αSyn. Although the presence of RER1 in human LBs was observed, co-IP assay showed no direct interaction between RER1 and αSyn (**S1 Fig**). Instead, we found association of RER1 with the E3 ligase NEDD4, indicating that the effects of RER1 on αSyn might be mediated by NEDD4.

NEDD4 ubiquitinates αSyn and has been implicated in regulating αSyn levels [48]. However, instead of targeting αSyn to the proteasome, NEDD4 appears to promote αSyn degradation via the endosomal-lysosomal pathway. Interestingly, we demonstrate that mutation of the only lysine (K80R) in the NAC region had no effect on RER1-mediated reduction of αSyn. Although αSyn has several other lysine residues that can be ubiquitinated, these findings suggest the possibility that RER1 effects on αSyn may occur via ubiquitin-independent proteasomal degradation. Indeed, there is evidence that some proteins including αSyn may be degraded in an ubiqutin-independent manner via the 20S proteasome [66,83,84]. Another possibility is that NEDD4 might instead have a role in ubiquitination and degradation of RER1 similar to the role of the known E3 ligase for RER1, synoviolin [30,31].

Further study is clearly needed to identify other UPS-related proteins that may interact with and regulate RER1 function. Although diverse protein-degradation pathways have been suggested to lower αSyn levels, the effects of regulating each of these pathways on αSyn pathology is still under debate. Our data demonstrate that RER1 and associated Golgi-ER retrieval mechanisms may play a pivotal role in regulating αSyn levels, accumulation, and associated toxicity. Additional studies are needed to determine the effects of RER1 on the fibrillization and toxicity of αSyn *in vivo*. As altered αSyn levels are linked to the development of PD and other LB-related diseases, further elucidating the mechanism of RER1 effects on αSyn is critical and may yield potential targets for therapeutic modulation aimed at mitigating αSyn-associated pathology.

## Supporting information

S1 Fig Co-immunoprecipitation studies do not demonstrate interaction between RER1 and αSyn. HEK293 cells were co-transfected with RER1 and wild type human αSyn. At 48 hours post transfection, lysates were collected and immunoprecipitated with either RER1 or αSyn antibodies (BD Biosciences). Western blots incubated with αSyn (top) or RER (bottom) antibodies show lack of co-immunoprecipitation. IP, immnopreciptation; PreImm, pre-immune absorption.
(PDF)
