# Supplementary material for: The ER retention protein RER1 promotes alpha-synuclein degradation via the proteasome
Source: PLoS One. 2017 Sep 6;12(9):e0184262. doi: 10.1371/journal.pone.0184262 (PMC5587320; doi:10.1371/journal.pone.0184262)
Supplement: S1 Fig — HEK293 cells were co-transfected with RER1 and wild type human αSyn. At 48 hours post transfection, lysates were collected and immunoprecipitated with either RER1 or αSyn antibodies (BD Biosciences). Western blots incubated with αSyn (top) or RER (bottom) antibodies show lack of co-immunoprecipitation. IP, immnopreciptation; PreImm, pre-immune absorption. (PDF) [file pone.0184262.s001.pdf]

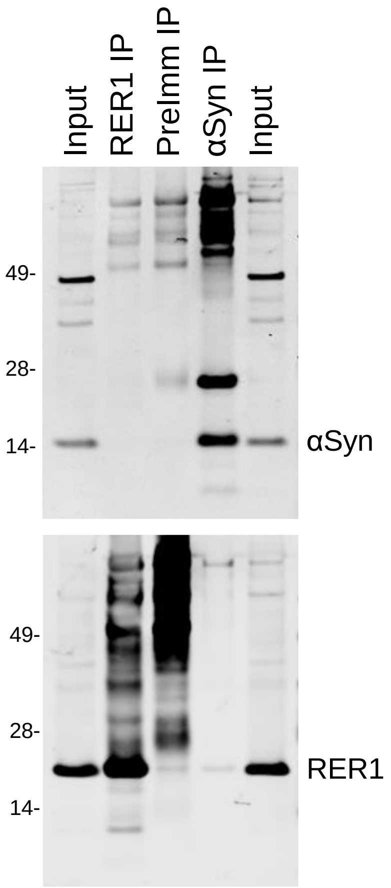

Figure S1. Co-immunoprecipitation studies do not demonstrate interaction between RER1 and αSyn. HEK293 cells were co-transfected with RER1 and wild type human αSyn. At 48 hours post transfection, lysates were collected and immunoprecipitated with either RER1 or αSyn antibodies (BD Biosciences). Western blots incubated with αSyn (top) or RER (bottom) antibodies show lack of co-immunoprecipitation. IP, immnopreciptation; PreImm, pre-immune absorption.
